# Targeted Quantification of the Lysosomal Proteome in Complex Samples

**DOI:** 10.3390/proteomes9010004

**Published:** 2021-01-26

**Authors:** Peter Mosen, Anne Sanner, Jasjot Singh, Dominic Winter

**Affiliations:** Institute for Biochemistry and Molecular Biology, Medical Faculty, University of Bonn, 53115 Bonn, Germany; pmos@uni-bonn.de (P.M.); anne.sanner@uni-bonn.de (A.S.); jsin@uni-bonn.de (J.S.)

**Keywords:** targeted proteomics, lysosomes, parallel reaction monitoring, data-independent acquisition, label-free quantification

## Abstract

In eukaryotic cells, lysosomes play a crucial role in the breakdown of a variety of components ranging from small molecules to complex structures, ascertaining the continuous turnover of cellular building blocks. Furthermore, they act as a regulatory hub for metabolism, being crucially involved in the regulation of major signaling pathways. Currently, ~450 lysosomal proteins can be reproducibly identified in a single cell line by mass spectrometry, most of which are low-abundant, restricting their unbiased proteomic analysis to lysosome-enriched fractions. In the current study, we applied two strategies for the targeted investigation of the lysosomal proteome in complex samples: data-independent acquisition (DIA) and parallel reaction monitoring (PRM). Using a lysosome-enriched fraction, mouse embryonic fibroblast whole cell lysate, and mouse liver whole tissue lysate, we investigated the capabilities of DIA and PRM to investigate the lysosomal proteome. While both approaches identified and quantified lysosomal proteins in all sample types, and their data largely correlated, DIA identified on average more proteins, especially for lower complex samples and longer chromatographic gradients. For the highly complex tissue sample and shorter gradients, however, PRM delivered a better performance regarding both identification and quantification of lysosomal proteins. All data are available via ProteomeXchange with identifier PXDD023278.

## 1. Introduction

Lysosomes are membrane-bound organelles, which are well-known as the main degradative compartment of eukaryotic cells [1]. They fulfil a crucial function for the breakdown of a variety of cellular components and the recycling of their building blocks. This is achieved by ~60 hydrolases and ~40 transporters residing in the lysosomal lumen and membrane [2]. The proper function of these hydrolases is crucial for cellular homeostasis, as exemplified by the detrimental consequences of lysosomal enzyme malfunction. Mutations resulting in their altered activity, stability, or subcellular distribution can result in the accumulation of their respective substrates within lysosomes, interfering with the correct function of the organelle. Impaired lysosomal function is the primary cause of a group of ~70 inherited rare genetic diseases, so-called lysosomal storage disorders (LSDs), which frequently result in neurodegeneration, metabolic dysfunction, impaired development, and premature death [3]. To date, therapies exist only for a handful of LSDs and those available are almost exclusively symptomatic [3,4,5].

While the connection between lysosomal dysfunction and LSDs has been known for decades, altered lysosomal or lysosome-associated proteins have recently been shown in an increasing number of studies to be involved in more common conditions, increasing the public interest in this organelle. This includes, but is not limited to, cancer [6], neurodegenerative disorders [7], and cardiovascular diseases [8]. As part of this development, the view on lysosomes as unregulated cellular waste bags, which persisted for decades, is currently transitioning towards highly mobile organelles that act as a major regulatory hub of cellular metabolism. In recent years, lysosomes have been shown to vary in their properties, to be actively transported, to interact with other organelles, and to respond to various cellular and environmental stimuli with the help of an extensive network of proteins [2,9,10]. This involves several key players regulating cellular growth and energy metabolism, such as the mechanistic target of rapamycin complex 1 (mTORC1) or the AMP-dependent kinase (AMPK), which are activated at the lysosomal surface [11].

These emerging roles of lysosomes have led to an increasing interest in the analysis of lysosomal proteins. For the unbiased characterization of large numbers of proteins, mass spectrometry (MS)-based proteomics is currently the method of choice, as it allows for the identification, quantification, and characterization of thousands of proteins from a given sample [12]. To date, ~740 proteins have been assigned in one way or the other to lysosomes, ~300 of which are either located in the lysosomal lumen, at the lysosomal surface, or directly interact with it [13]. 

Lysosomal proteins are typically of low abundance and therefore frequently not covered in DDA whole proteome shotgun analyses. The most common way to increase the coverage of lysosomal proteins is lysosome enrichment, resulting in a reduced sample complexity and therefore facilitating their analysis. Several lysosome enrichment methods are currently available, the most common of which are based either on density gradient centrifugation, superparamagnetic iron oxide nanoparticles (SPIONs) in combination with magnetic columns, or the immunoaffinity enrichment of tagged lysosomal proteins [14]. While all of these approaches allow for a certain degree of enrichment, they come with restrictions that limit the samples they can be applied to. While density gradient centrifugation can be performed for virtually any starting material, recovery is low and high amounts of contaminating organelles, mostly mitochondria, are included in the lysosome-containing fractions [14]. The use of SPIONs, which are taken up by unspecific fluid phase endocytosis and delivered to the lysosomal compartment through the endocytic pathway [15], is limited to cells grown in culture which actively perform fluid phase endocytosis. Furthermore, with this approach, only those lysosomes can be isolated that receive cargo from late endosomes, which may be affected when studying models of LSDs with impaired endosome-lysosome fusion. For the immunoaffinity enrichment of lysosomes through tagged membrane proteins [16,17], a fusion protein has to be stably expressed in cells or animals, requiring the generation of the respective organism. Furthermore, only lysosomes expressing the protein of choice are covered, which may result in a selection bias, and the overexpression of the tagged protein may influence lysosomal properties. For all approaches, millions of cells or milligram amounts of tissue are needed as starting material, excluding low-abundant samples from these analyses. As many LSDs affect distinct populations of cells, and the majority of LSDs can only be simulated in animal models, these limitations stall the proteomic investigation of LSDs, as it is frequently not possible to obtain lysosome-enriched fractions in sufficient quantities. 

The need for enrichment of lysosomal proteins arises from the limitations of untargeted data-dependent acquisition (DDA)-based acquisition strategies, as highly abundant peptides prevent the fragmentation, and therefore identification, of those originating from low-abundant lysosomal proteins. Therefore, a promising alternative for the characterization of lysosomal proteins from small amounts of complex samples are targeted proteomics strategies. Currently, two major approaches for targeted proteomics are applied. On the one hand, previously defined peptides are fragmented in single, multiple, or parallel reaction monitoring (SRM, MRM, PRM) experiments, and abundance is determined based on the intensity of their fragment ions [18]. On the other hand, unbiased fragmentation of pre-determined m/z windows is performed in data-independent acquisition (DIA) approaches, and the abundance of the respective peptides is determined from unique fragment ions identified in mixed MS/MS spectra [19]. In comparison to DDA-based label-free quantification strategies, PRM and DIA approaches offer increased sensitivity and reproducibility for low-abundant peptides in complex samples [20,21,22], making them ideal candidates for the analysis of lysosomal proteins from cell or tissue samples without prior enrichment. 

So far, to our knowledge, targeted approaches have not been frequently used for the investigation of the lysosomal proteome. PRM was applied in a few studies for the investigation of selected lysosomal proteins (e.g., [23,24,25]), while only DIA approaches have been used for the analysis of the whole lysosomal proteome, so far solely investigating lysosome-enriched samples [14,16,26]. While it was reported that DIA is able to identify and quantify > 10,000 proteins within a single run [27], the reproducible quantification of lysosomal proteins suffers in highly complex samples and the achievable performance in whole cell lysates is significantly lower compared to lysosome-enriched fractions [14].

In the present study, we compared DIA and PRM for the analysis of the lysosomal proteome from samples of different complexities. We investigated lysosome-enriched fractions, as well as whole cell and liver lysate, and systematically compared the performance of DIA and PRM. While we could detect lysosomal proteins with both approaches in all sample types, and DIA identified higher numbers for most samples, PRM showed a better performance in liver lysate allowing for the detection of quantitative changes which were not identified by DIA.

## 2. Material and Methods

### 2.1. Cell Culture Experiments and Sample Lysis

All cell culture experiments were performed under a sterile hood and all solutions were pre-warmed to 37 °C. Mouse embryonic fibroblasts (MEFs) were cultured at 37 °C and 5% CO_2_ in Dulbecco’s modified eagle medium (DMEM), supplemented with 10% (*v*/*v*) fetal calf serum (FCS), 100 IU/mL penicillin, and 100 µg/mL streptomycin. For the generation of MEF whole cell lysate samples, 1.5 × 10^6^ cells each were seeded on three 15 cm plates and cultivated for 72 h. The cells were washed once with 5 mL of ice-cold 1× phosphate-buffered saline (PBS), scraped in 600 µL of ice-cold PBS, and collected in a 1.5 mL microtube. Cells were pelleted by centrifugation at 1000× *g* and 4 °C for 4 min, the supernatant was discarded, and the cell pellet was re-suspended in 600 µL lysis buffer (4% SDS, 100 mM HEPES pH 7.5). Subsequently, the cell suspension was incubated at 95 °C for 10 min followed by sonication using a Ultrasonics Sonifier 250 (Branson, Danbury, CT, USA) at a duty cycle of 60% and an output of 6 for 90 s. Samples were centrifuged at 20,000× *g* and RT for 30 min and the clear supernatants were transferred to new microtubes. 

Lysosome isolation was performed from MEF cells using SPIONs as described elsewhere [26]. In brief, cells were cultivated in DMEM with 2.5% FCS for 72 h (3 × 10^6^ cells per 10 cm dish), 1 mL of magnetite solution (EndoMAG40, Liquids Research, North Wales, UK) was added to each plate, and the cells were incubated for 24 h (pulse period). Subsequently, the cells were washed twice with pre-warmed PBS, fresh DMEM (10% FCS) was added, and the cells were incubated for 24 h (chase period). Cells were washed with ice-cold PBS and harvested using a cell scraper in 2 mL lysosome isolation buffer (250 mM sucrose, 10 mM HEPES pH 7.4, 1 mM CaCl_2_, 15 mM KCl, 1 mM MgCl_2_, 1.5 mM MgAc, 1 mM dithiothreitol (DTT), 1x cOmplete EDTA-free protease inhibitor cocktail (Roche Diagnostics, Mannheim, Germany)). Plasma membranes were disrupted using a dounce homogenizer, and lysosomes were enriched using Miltenyi LS columns (Miltenyi Biotech, Auburn, CA) and eluted using a plunger. 

### 2.2. Preparation of Mouse Liver Samples

Mice were handled in accordance with local regulations concerning the welfare of animals. Three months-old male C57BL/6 mice were sacrificed by cervical dislocation, the liver was extracted, and snap-frozen in liquid nitrogen. The frozen tissue was chopped into small pieces using a razor blade, and 1 mL of lysis buffer (4% SDS, 100 mM HEPES pH 7.5) was added. The sample was incubated for 10 min at 95 °C and sonicated using an Ultrasonics Sonifier at a duty cycle of 60% and an output of 6 for 90 s. Subsequently, the samples were again incubated at 95 °C for 10 min, centrifuged at 20,000× *g* and RT for 30 min, and the clear supernatants were transferred to new microtubes. 

### 2.3. Sample Preparation for Mass Spectrometry

The protein concentration of all samples was determined using the DC Protein Assay (Bio-Rad Laboratories, CA, USA). For MEF whole cell lysate and liver samples, 100 µg of protein were used for each replicate while 20 µg were used for lysosome-enriched fractions. Sample volumes were adjusted to 200 µL using HPLC-grade water and proteins were precipitated by addition of 1 mL ice-cold chloroform/methanol (2:1 *v*/*v*), vigorous vortexing, and centrifugation at 20,000× *g*, 4 °C for 1 h. The liquid phases were discarded, the protein pellets washed once with 1 mL of ice-cold methanol, and centrifuged at 20,000 × *g*, 4 °C for 15 min, followed by the removal of methanol. Protein pellets were air-dried and solubilized in 1% RapiGest (Waters, Milford, MA, USA), 0.1 M NH_4_HCO_3_ pH 7.8 at 95 °C for 10 min. Subsequently, samples were diluted 1 to 5 with 0.1 M NH_4_HCO_3_ and trypsin (Promega, Mannheim, Germany) was added at an enzyme-to-substrate ratio of 1:500, followed by incubation at 37 °C, 800 rpm in a thermomixer for 45 min. Proteins were reduced using DTT (5 mM final concentration) at 56 °C for 30 min and alkylated with acrylamide (20 mM final concentration) for 30 min at RT, followed by quenching of the reaction through the addition of 5 mM DTT. Finally, trypsin was added at an enzyme-to-sample ratio of 1:50 and the RapiGest concentration adjusted to 0.1% using 0.1 M NH_4_HCO_3_. Proteins were digested overnight at 37 °C, and on the following day, RapiGest was hydrolyzed by addition of 1% TFA (final concentration) and incubation in a thermomixer at 800 rpm, 37 °C for 30 min. Hydrolyzed RapiGest was precipitated by centrifugation at 20,000× *g*, RT for 10 min and the supernatants were desalted using Oasis HLB cartridges (Waters) as described elsewhere [28]. Briefly, cartridges were equilibrated with 70% ACN, 0.1% acetic acid (AA), washed with 0.1% AA, and the sample was loaded. Subsequently, cartridges were washed with 0.1% AA and peptides were eluted sequentially with 30%, 50%, and 70% ACN, 0.1% AA. Eluate fractions were pooled and the combined samples dried in a vacuum centrifuge. Dried peptides were re-suspended in 5% ACN, and the peptide concentration was determined using the Quantitative Fluorometric Peptide Assay (ThermoFisher Scientific, Waltham, MA, USA), and the peptides were dried again.

### 2.4. LC-MS/MS Analysis

All analyses were performed using a Dionex Ultimate 3000 nano-UHPLC system coupled to an Orbitrap Fusion Lumos mass spectrometer (both Thermo Fisher Scientific). Analytical columns were produced in-house as follows: spray tips were generated with a P-2000 laser puller (Sutter Instruments, Novato, CA) from 360 µm outer diameter and 100 µm inner diameter fused silica capillaries and packed to a length of 40 cm with 3 µm ReprosilPur AQ C_18_ particles (Dr. Maisch, Ammerbuch-Entringen, Germany). Dried peptides were reconstituted in 5% ACN, 5% formic acid (FA), and 1 µg was loaded together with 750 fmol of internal retention time standards (iRTs, Biognosys, Schlieren, Switzerland) to the analytical column at a flow rate of 600 nl/min with 100% solvent A (0.1% FA in water) for 25 min. Peptides were eluted with 60, 120, and 240 min linear gradients from 5–35% solvent B (90% ACN, 0.1% FA) at a flow rate of 300 nl/min. For parallel reaction monitoring (PRM) measurements, precursor masses were selected from a previously recorded dataset (Table S1, [26]) while the spectral library for data-independent acquisition (DIA) analyses was generated using 240 min data-dependent acquisition (DDA) runs. In these analyses, survey spectra were acquired with a mass range of m/z 350–1200 at a resolution of 60,000 and an AGC target setting of 4 × 10^5^ The most abundant precursor ions (charge states of 2–4) were isolated using the quadrupole (isolation width of m/z 1.6), and fragmented by HCD with a collision energy of 27 in the top speed mode (cycle time of 3 sec). Fragment ion spectra were acquired in the Orbitrap mass analyzer at a resolution of 30,000 and fragmented ions were excluded from further fragmentation for 120 s. For DIA analyses, one MS scan was performed with a mass range of m/z 350–1200, a resolution of 120,000, a maximum injection time of 20 ms, and an AGC target setting of 5 × 10^5^. The MS scan was followed by static DIA MS/MS scans, covering the same m/z range with an overlap of m/z 0.5, with the following gradient lengths/ scan numbers/ isolation windows/ cycle times: 60 min/ 24 scans/ m/z 35.9/ 2.34 s; 120 min/ 36 scans/ m/z 24.1/ 3.44 s; 240 min/ 58 scans/ m/z 15.2/ 5.45 s). The HCD collision energy was set to 27% and DIA MS/MS scans were acquired in the Orbitrap with a resolution of 30,000, a maximum injection time of 60 ms, and an AGC target setting of 1 × 10^6^. For PRM analyses, MS spectra were acquired with a mass range of m/z 300–1500 at a resolution of 60,000, a maximum injection time of 118 ms, and an AGC target setting of 4 × 10^5^. Peptides were isolated in the quadrupole with an isolation width of m/z 1.2 and fragmented by HCD with a collision energy of 27%. MS/MS scans were acquired in the Orbitrap mass analyzer with a mass range of m/z 200–2000, a resolution of 30,000, a maximum injection time of 54 ms and an AGC target setting of 5 × 10^4^.

### 2.5. Data Analysis

For DIA library generation, DDA *.raw files were analyzed with the Pulsar search engine integrated in Spectronaut (Version: 14.7.20, Biognosys) (1). Uniprot *Mus musculus* (release date: 09.09.2019 with 17,023 entries), in combination with a database containing common contaminants, was used for database searching with Spectronaut standard settings [29]. In brief, cleavage by trypsin with up to two missed cleavage sites was defined, propionamide (cysteine) was set as fixed and oxidation (methionine) as variable modification, and three to six fragment ions were selected for library generation, dependent on the intensity of the respective peptide. The high-precision iRT concept (dynamic) was applied for retention time alignment. Matching of mass tolerances for precursors, fragment ions, as well as peak extraction windows were determined automatically by Spectronaut. Only MS precursor information was utilized for peak detection, and interference correction was enabled. Global normalization was performed for individual runs based on the median abundance. Data were filtered with a 1% FDR cut off on the precursor and protein level (q-value < 0.01) [30]. *p*-value determination and unsupervised clustering were performed with the post-analysis pipeline of Spectronaut applying default parameters (distance metric: Manhattan Distance; linkage strategy: Ward’s method; multiple testing correction: Storey’s method).

For PRM analyses, a spectral library was generated using a subset of our previously published DDA dataset [26] with Skyline [31], applying a cut-off score of 0.95. Ambiguous peptide matches were excluded, and the library was filtered for peptides which were previously manually selected to be included in the assay (Table S1). For analysis of PRM data, *.raw files were loaded into Skyline daily version 20.2.1.315. Automated fragment ion selection by Skyline was utilized (6 ions/peptide) with the exception of the peptides with the sequence SLQPLYR and GSFSLSVR, for which only 5 fragment ions matched, using the following criteria: maximum mass error of 10 ppm for MS and MS/MS ion trace filtering (centroid mode) and charge states of 1+/2+ for b- and y-ions as well as 2+/3+ for precursor ions. Integration boundaries of iRT peptides were inspected manually and corrected, if necessary. Experimental data were only reviewed when Skyline reported a peak truncation, and peptides with truncated peaks or no MS/MS signal were excluded from further analysis. Peptide-centric reports were exported and further processed in MS Excel. For peptide and protein quantification, the summed area under the curve (AUC) of fragment ions was used. For all analyses, only peptides with quantitative values in all three replicates were considered.

## 3. Results and Discussion

We showed previously that the analysis of lysosome-enriched fractions with DIA allows for a superior performance compared to DDA measurements in a reduced amount of time [26]. When we investigated the lysosomal proteome in samples of higher complexity (such as whole cell lysates); however, we observed that the number of lysosomal proteins that can be reproducibly identified and quantified was markedly lower, indicating a reduced performance in such samples [14]. This is most likely due to the fact that co-fragmenting peptides increase the complexity of MS/MS spectra, which results in a decreased performance for the quantification of lysosomal proteins, as they are of low abundance relative to the whole proteome. In theory, PRM approaches should be superior in this aspect, as only a small m/z window, that is specific for the individual peptide, is fragmented. 

**Figure 1:**
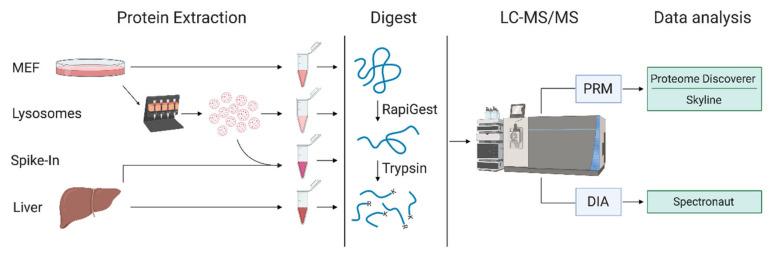
Workflow for sample preparation and analysis. For each sample type, proteins were extracted and digested in three experimental replicates and analyzed by LC-MS/MS using either DIA or PRM with different gradient lengths. MEF: mouse embryonic fibroblasts; DIA: data independent acquisition; PRM: parallel reaction monitoring; K: lysine; R: arginine. Created with Biorender.com.

In order to determine which strategy is best-suited for the MS/MS-based quantification of the lysosomal proteome in samples with different complexities, we compared DIA- and PRM-based quantification (Figure 1). Initially, we defined a highly reproducible lysosomal proteome from a dataset generated previously by our group, comprising 39 DDA LC-MS/MS runs of lysosome-enriched fractions from MEFs [26]. From these data, we only considered proteins which were assigned to the lysosomal compartment based on gene ontology (GO) and Uniprot categories, and which were detected in ≥ 75% of LC-MS/MS runs with ≥ 2 unique peptides, resulting in a final list of 374 proteins (Table S1). For the comparison of DIA and PRM, we used a lysosome-enriched fraction from MEFs (LEF) as benchmark samples, as it contains the highest percentage of lysosomal proteins. Furthermore, we used MEF whole cell lysate (MWCL) as well as liver tissue lysate (LTL), representing samples of increasing complexity. We performed all experiments in triplicates with independent experimental replicates for each measurement. 

### 3.1. Gradient Length and Sample Complexity Affect Lysosomal Protein Quantification by DIA

Initially, we analyzed all three sample types with different gradient lengths (60 min, 120 min, and 240 min) by DIA. We adjusted the width of DIA fragmentation windows depending on the gradient length in order to allow for a similar number of data points across chromatographic peaks of the individual gradients. Consequently, a shorter gradient resulted in a larger m/z window and vice versa, influencing the number of co-fragmented precursor ions. To assess performance of the individual methods, we evaluated both the numbers of total proteins, and those previously reported to be lysosomal (Table S1) that were identified in each run (Table S2). 

**Figure 2:**
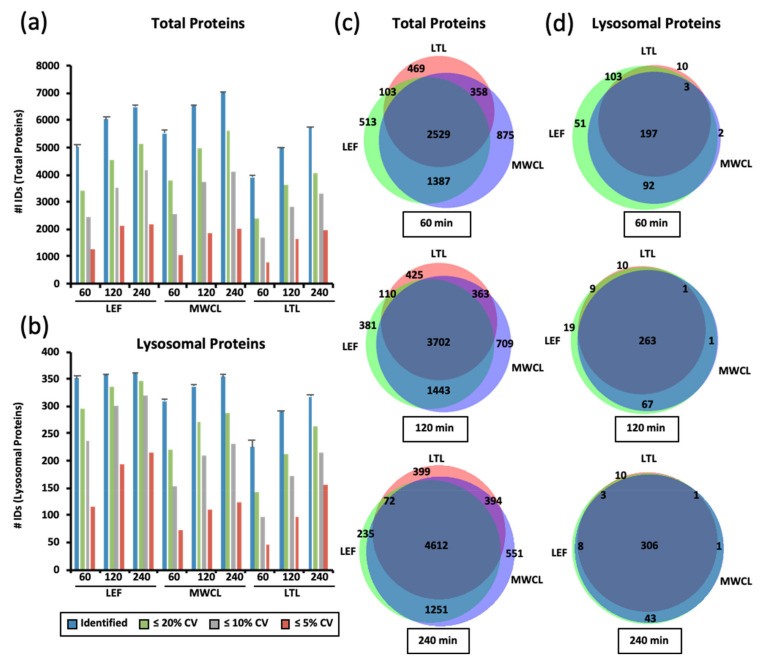
Identification of total and lysosomal proteins in DIA experiments. (**a**,**b**) Numbers of identified total proteins (**a**) and those known to be located at/in the lysosome (**b**). Shown are average values (n = 3) for the analysis of lysosome-enriched fractions of MEF (LEF), MEF whole cell lysate (MWCL), and liver tissue lysate (LTL) with three different gradient lengths. Total IDs as well as those quantified with a CVs ≤ 20%, 10%, and 5% are shown. (**c,d**) Overlap in protein identification for total proteins (**c**) and those known to be located at the lysosome (**d**) for proteins identified in all three replicates of LEF, MWCL, and LTL samples analyzed with different gradient lengths. Venn diagrams were generated with the tool BioVenn [32]. LEF: lysosome-enriched fractions from mouse embryonic fibroblasts; MWCL: mouse embryonic fibroblast whole cell lysates; LTL: whole liver tissue lysate; CV: coefficient of variation; ID: number of identified proteins.

We found highest total protein numbers in the MWCL, followed by the LEF, and the LTL (Figure 2a). While we observed a steady increase in the number of identified total proteins from 60 min to both 120 min and 240 min gradients for MWCL (increase of 19% and 28%) and LTL (increase of 28% and 46%) samples, the numbers of IDs detected in the LEF only increased from 60 min to 120 min gradients (increase of 30%). When considering only lysosomal proteins, we identified, as expected, highest numbers in the LEF, followed by MWCL and LTL. While the latter two showed a similar correlation of gradient length and protein identifications, the LEF produced virtually constant numbers for all gradients and only CV values improved. The differences in identifications were particularly pronounced when considering lysosomal proteins quantified with < 5% CV in the 60 min gradient analyses, 116 of which were found in the LEF but only 45 in the LTL (Figure 2b). 

It was quite surprising to us that we identified the highest number of proteins in the MWCL, as the LTL should theoretically be the most complex sample. A possible explanation for this observation is that the LTL contains a certain number of highly abundant proteins, which account for a larger percentage of the sample than highly abundant MWCL and LEF proteins. Consequently, in LTL the remaining proteins present a smaller fraction of the total sample. As the C-trap — which is used for ion storage prior to injection into the Orbitrap — has a limited capacity [33], this results in reduced fragment ion intensities for the lower abundant proteins, which are not sufficient for identification/quantification. Furthermore, the highly abundant fragment ions from these proteins dominate the DIA MS/MS spectra, resulting in reduced detection of co-fragmented lower abundant peptides.

When considering the increase in identification of total unique proteins with increasing analysis time, each sample contributed a distinct population (Figure 2c). Lysosomal protein identifications, on the other hand, were very similar between the samples, and for 240 min gradients the majority was identified at least in both the MWCL and the LEF (Figure 2d). These results imply that short gradients suffice to achieve a good coverage of lysosomal proteins in LEFs, while longer gradients are needed when MWCL and LTL are analyzed. 

While this confirms that lysosomal proteins are more abundant in lysosome-enriched fractions, which was certainly expected, it also shows that the detection of such lower abundant proteins in DIA analyses suffers from sample complexity. As our DIA analyses were performed with different m/z windows for the different gradient lengths, this effect is most likely related to the number of co-fragmented precursor ions and the resulting MS/MS spectrum complexity. The bigger the fragmentation window is, the more peptides are co-fragmented, and consequently the fragment ions of the lower abundant lysosomal proteins are identified with a lower efficiency.

### 3.2. Variation of Protein Abundance and Variance between Sample Types in DIA Analyses

**Figure 3:**
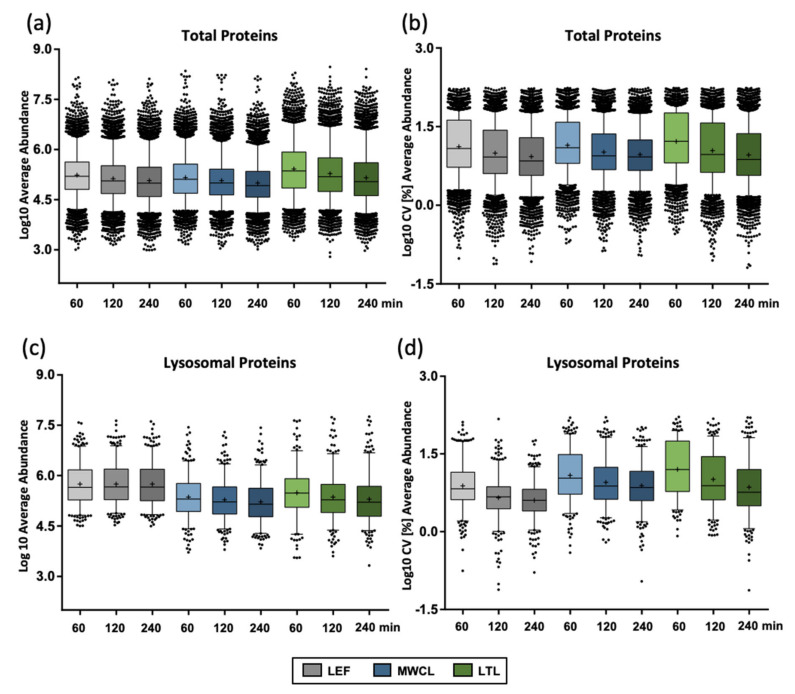
Reproducibility of protein abundance in DIA analyses. (**a**) Protein abundance for total proteins identified in individual samples with different gradient lengths. (**b**) CV values for total proteins identified in individual samples with different gradient lengths. (**c**) Protein abundance for lysosomal proteins identified in individual samples with different gradient lengths. (**d**) CV values for lysosomal proteins identified in individual samples with different gradient lengths. Shown are combined values from 3 replicates, the median is indicated by a line, while the average is marked with a “+”. LEF: lysosome-enriched fractions from mouse embryonic fibroblasts; MWCL: mouse embryonic fibroblast whole cell lysates; LTL: whole liver tissue lysate; CV: coefficient of variation.

To further follow up on this effect, we investigated the protein abundances for the individual samples utilizing median-normalized AUCs (Figure 3a). Confirming our previous assumption, the liver lysate resulted in the highest average abundance (1.6- and 1.8-fold higher compared to the lysosome-enriched fractions and MEF lysate, respectively, for 60 min gradients) and the largest number of highly abundant proteins (36 proteins compared to 11 and 12 with log10 values > 7.5 for LEF and MWCL, respectively). Average protein abundance correlated inversely with the number of protein identifications, with highest values in the shortest gradient, irrespective of the sample type. For lysosomal proteins, we observed highest average abundances in the LEF (1.8- and 2.4-fold higher compared LTL and MWCL for 60 min gradients) and, unlike the total protein identifications, no decrease in abundance with increasing gradient length (Figure 3c). Average CV values, however, behaved similarly for all types of proteins (Figure 3b,d).

**Figure 4:**
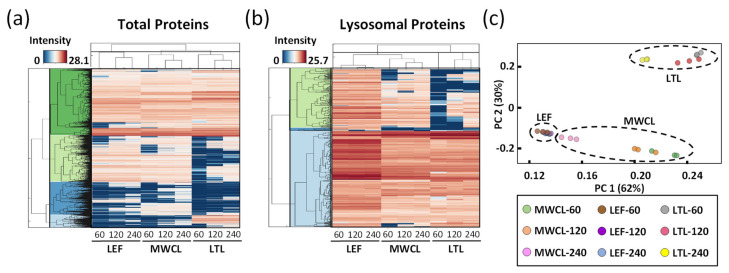
Global analysis of DIA datasets. (**a**,**b**) Unsupervised clustering of average abundances (columns) of LEF, MWCL, and LTL for three different gradient lengths (60, 120 and 240 min) for total proteins identified ((**a**), n = 7145) and lysosomal proteins ((**b**), n = 314). The color code indicates the normalized intensity of the individual proteins. (**c**) Principal component analysis (PCA) for all analyses with two defined standardized principal components (PC1 and PC2). LEF: lysosome-enriched fractions from mouse embryonic fibroblasts; MWCL: mouse embryonic fibroblast whole cell lysates; LTL: whole liver tissue lysate.

To visualize the differences of the individual datasets on a global scale, we generated heatmaps for the average abundances of total and lysosomal proteins, clustered in a row- and column-wise manner (Figure 4a,b). For both analyses, we observed distinct protein populations which formed individual clusters, based on their abundance in the respective sample types and gradient lengths. In most cases, gradient length played a decisive role, while the highest differences existed for the LTL relative to the other samples. For the majority of known lysosomal proteins, we detected a higher abundance in the LEF relative to the MWCL and the LTL, while certain proteins were exclusively identified in the LEF. We also identified, however, some clusters with a higher abundance in MWCL and LTL, implying that either not all lysosomal proteins were recovered efficiently in the lysosome-enrichment step, or that a certain population of these proteins was located in a different cellular compartment. 

Finally, we assessed the global variability between the datasets by principal component analysis (PCA, Figure 4c). The two main principal components (PC1 and PC2), which are responsible for 62% and 30% of the variance in the dataset, allowed for a good separation of the samples. As individual replicates of the same sample type and gradient length clustered closely together, the main variance in the dataset (PC1) can be explained based on the difference of the sample type itself. However, especially for the MWCL, the 240 min gradient data behaved significantly differently than those acquired with other gradients, being actually closer to the LEF. This relates most likely to the fact that the LEF originated from MEFs and that proteome coverage in the 240 min gradient increased to such an extent that very similar proteins were identified (Figure 2c). 

### 3.3. PRM Assay Development

**Figure 5:**
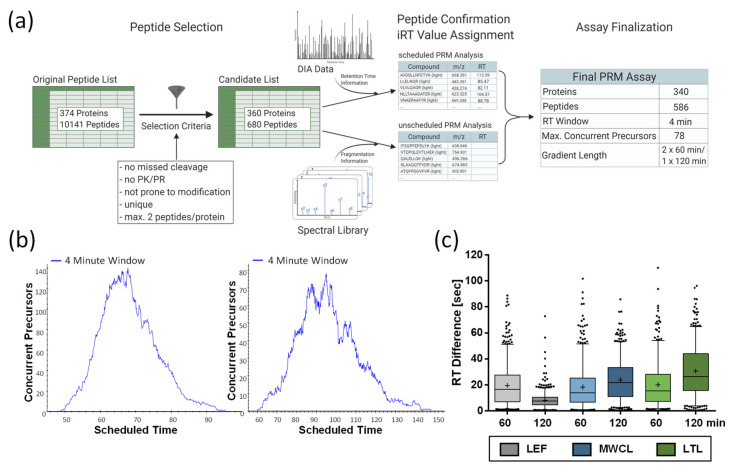
Establishment of PRM assay. (**a**) Workflow for the development of the PRM assay. Created with Biorender.com. (**b**) Distribution of concurrent precursor elution in 60 min and 120 min gradients. (**c**) Differences between predicted and experimentally determined retention times for individual measurements. Shown are combined values of three replicates and the mean (+), median (line), and interquartile range are indicated. LEF: lysosome-enriched fractions from mouse embryonic fibroblasts; MWCL: mouse embryonic fibroblast whole cell lysates; LTL: whole liver tissue lysate; CV: coefficient of variation; RT: retention time.

For the 374 proteins included in our lysosomal proteome reference list (Table S1), 10,141 unique peptides were identified in the course of our previous analysis [26]. We narrowed down the list of putative peptides by excluding those identified with variable modifications, missed cleavage sites, or containing the amino acid combination PK or PR (as proline residues interfere with tryptic cleavage). These criteria were fulfilled by 3816 peptides representing 367 proteins. Based on the average signal intensities in this dataset, we considered the two most abundant peptides for each protein, resulting in a final list of 680 peptides, as for some proteins only a single peptide fulfilled our criteria (Figure 5a, Table S1).

For PRM assay scheduling, we extracted the peptides’ retention time information from the 120 min DIA runs of the LEF, followed by the refinement of the assay by PRM analysis of the same sample, including high-precision indexed retention time (iRT) standards [29]. After analysis with an initial scheduled PRM assay (15 min retention time windows), we performed unscheduled PRM runs for those peptides that were not detected in these initial analyses. Finally, we combined the acquired retention times of all peptides that we were able to detect with distinct fragment ion signatures, and created an iRT-normalized library. This resulted in a final assay comprising 586 peptides from 340 lysosomal proteins. For the analysis of acquired PRM data with Skyline [31], we built a reference spectral library from our previously measured DDA dataset of the LEF [26]. Finally, we generated two assays utilizing 4 min retention time windows. In one assay, all peptides were analyzed in a single 120 min gradient, while the other assay consisted of two 60 min gradients. This was necessary, as the high number of concurrent precursors (up to 140) would have drastically reduced the number of data points for chromatographic peaks eluting in the middle of gradient (Figure 5b). For subsequent analyses, we determined iRT correction factors for the different sample types using DDA runs, and adjusted the scheduling accordingly. For data export from Skyline, a minimum number of six data points was defined. 

### 3.4. Gradient Length and Sample Type Affect Data Quality in PRM Analyses

Due to the high number of peptides, we defined parameters for the acceptance of PRM quantification data without manual inspection of each peptide. Initially, we assessed the difference between predicted and experimentally observed retention times. For the 60 min and 120 min gradients, we observed average peak widths of 21 sec and 25 sec, respectively, and an average retention time variability of ±15 sec, with a slightly lower average shift for the 60 min gradients (Figure 5c). LEF analyses with 120 min gradients presented with only ±8 sec an exception, which might be due to the fact that we performed the PRM assay retention time normalization with 120 min gradient measurements of the LEF, while the scheduling for MWCL and LTL was solely based on iRT predictions. 

**Figure 6:**
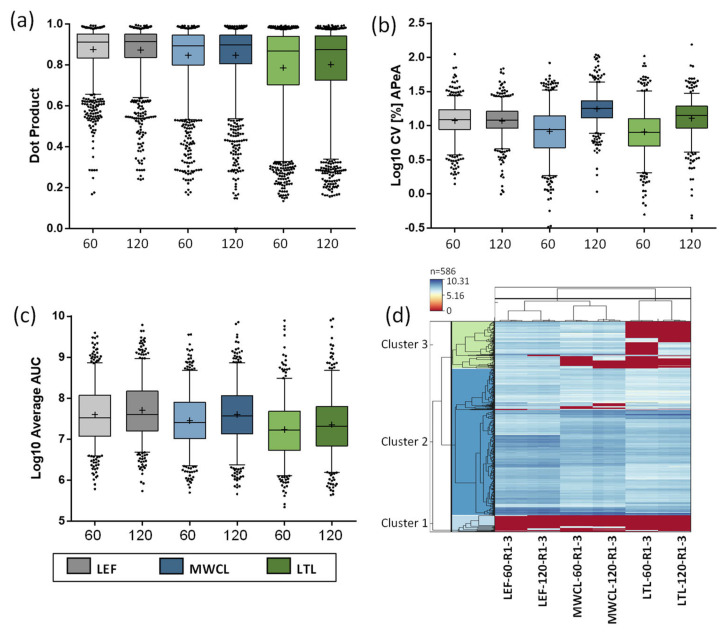
Characterization of data generated by PRM analysis. (**a**) Assessment of data quality in PRM analyses by Dot Product values. (**b**) Log10 CV values for the summed AUC of all targeted peptides. (**c**) Averaged log10-transformed summed peptide AUCs of the replicates (n = 3) across the different sample types and gradient lengths from PRM measurements. (a/b/c) Indicated are mean (+), median (line), and interquartile range. (**d**) Heatmap of the log10-transformed AUCs of all peptides covered by the PRM assay across all measurements. Each column contains data from one measurement and each row represents one peptide. Peptides are clustered if they exhibit similar trends across the samples. LEF: lysosome-enriched fractions from mouse embryonic fibroblasts; MWCL: mouse embryonic fibroblast whole cell lysates; LTL: whole liver tissue lysate; AUC: area under the curve; CV: coefficient of variation; APeA: average peptide abundance.

Next, we investigated the quality of acquired fragment ions for the individual peptides utilizing the dot product (dotP) value [34], which allows for correlation between the acquired spectrum and the spectral library (generated from our reference dataset [26]). Especially for the analysis of unfractionated highly complex samples, this allows to identify the impact of interfering ions that may result in false quantification results. Across all analyses, the average dotP value was > 0.85, indicating a good matching of our PRM data with the spectral library (Table S3). While we observed roughly similar dotP values for both the 60 min and 120 min assays, they decreased with sample complexity (Figure 6a). Compared to the LEF, which displayed the least variation, especially the LTL resulted in lower dotP values and higher variability. These findings imply a lower relative abundance of lysosomal proteins and an increase of interfering fragment ions in the MS/MS spectra for the more complex samples, which is also in agreement with the DIA data. 

Subsequently, we investigated the correlation of dotP values and numbers of fragment ions used for quantification of the different sample types and gradient lengths (Figure S1). Utilizing three to six fragment ions, we applied different dotP value thresholds and determined the number of peptides passing it. As expected, lower numbers of fragment ions resulted in more peptides passing the threshold at higher dotP values. This was especially true when dotP thresholds ≥ 0.9 were applied, as we observed a clear difference between the peptides identified with 3, 4, 5, or 6 fragment ions. For lower dotP values (0.7–0.8), this effect was far less pronounced. As already indicated by the average dotP values (Figure 6a), an inverse correlation with sample complexity could be observed. Based on these analyses, we defined 6 fragment ions per peptide with a dotP value of 0.7 as cut-off for the acceptance of quantification information from PRM data. 

For LEF data, this cut-off resulted in an acceptance rate of 92% of the peptides included in our assay for both gradients. The value for MWCL was 87% for both gradients and for LTL 73% and 78%, for the 60 min and 120 min gradient, respectively. Applying these cut-offs, we exported the data from Skyline and utilized them for all further analyses (Table S4). 

### 3.5. PRM Analysis of the Lysosomal Proteome

We initially investigated the reproducibility of quantification (Figure 6b). While CVs of the LEF analyses were similar for both gradients, the 120 min gradient resulted in consistently higher CVs for both MWCL and LTL. Surprisingly, the higher complexity samples resulted in a lower average CV than the LEF for the 60 min runs. A possible explanation for this observation is the lower sample amount utilized for LEF sample preparation (~20 µg) compared to MWCL and LTL (~100 µg), which may have resulted in a higher variability during pipetting and desalting. Subsequently, we calculated the average summed area under the curve (AUC) for each sample type and gradient length (Figure 6c). Interestingly, while we saw a higher summed abundance for the LEF in comparison to the other samples for 60 min gradients, the values were more similar for the 120 min analyses, especially for the comparison of LEF and MWCL. This could be related to the different numbers of data points acquired over the chromatographic peak as well as variances in peak width/shape between gradients. 

Finally, we assessed the overall correlation of the data in a heatmap, depicting the signal intensities of individual peptides in each sample and replicate, clustered in a row- and column-wise manner (Figure 6d). In general, the lysosomal peptides formed three distinct clusters. Two clusters showed similar expression levels (general high or low expression) in all samples, while the third cluster contained proteins that were detected with differing levels in the individual samples. In agreement with the DIA data, we observed subsets of peptides that were only found in the LEF, and were not detected in both the MWCL and LTL. Moreover, peptides existed that were detected both in LEF and MWCL, but not in LTL, and a small subset with higher abundance in LTL compared to the other samples. 

### 3.6. Comparison of DIA- and PRM-Based Quantification of the Lysosomal Proteome

**Figure 7:**
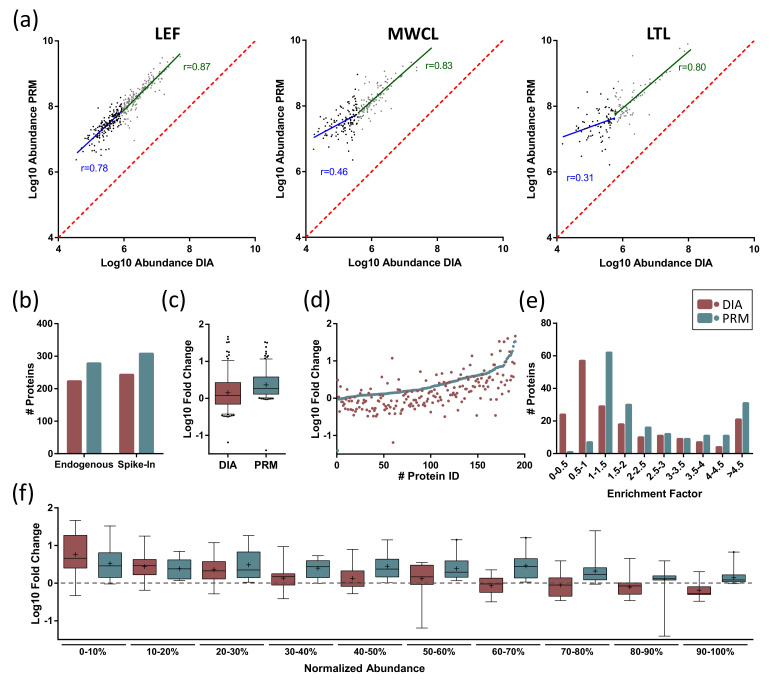
Comparison of DIA and PRM analysis of lysosomal proteins. (**a**) Correlation of normalized peptide signal intensities for DIA and PRM runs. Proteins are sorted based on their intensity in DIA measurements and grouped into two groups based on their intensity (upper/lower 50% of proteins). For each population, a linear regression analysis was performed and the respective correlation coefficient (r) is indicated. (**b**) Identification of proteins in LTL with and without spike-in of LEFs. (**c**,**d**) Fold change values for individual lysosomal proteins in LTL with spike-in of LEFs. (**e**) Frequency of proteins within distinct fold change quantiles for DIA and PRM data for ratios of LTL with/without spike-in of LEFs. (**f**) Protein fold change values for LTL with/without spike-in of LEFs. Proteins are grouped based on their abundance in the respective dataset relative to the highest/lowest-abundant protein. LEF: lysosome-enriched fractions from mouse embryonic fibroblasts; MWCL: mouse embryonic fibroblast whole cell lysates; LTL: whole liver tissue lysate.

In order to correlate the performance of DIA- and PRM-based quantification of the lysosomal proteome, we initially compared the data obtained from the individual datasets. When considering the average abundance and CVs of lysosomal proteins, we observed for all DIA analyses that longer gradients resulted in lower CV values but also lower abundances (Figure 3c,d), while for PRM both intensities and CVs (with the exception of the LEF data) increased with gradient length (Figure 6b,c). When considering how many lysosomal proteins were found in the individual analyses, we identified higher numbers for DIA, with the exception of LTL analyzed with 60 min gradients (Figure S2). To assess to what extend the abundances acquired with the individual approaches correlate, we extracted the AUCs of the peptides included in our PRM assay from the DIA dataset (60 min gradients for both approaches) and performed a direct comparison (Figure 7a). We observed for all three sample types that PRM resulted in higher signal intensities than DIA and that correlation of signal intensities was dependent on the abundance of the respective protein. We observed a good correlation for high-abundant proteins (upper 50% of DIA intensities) in all sample types. For low-abundant proteins (lower 50% of DIA intensities), we only detected a good correlation between DIA and PRM for the LEF. For the more complex samples, however, DIA seemed to underestimate high signal intensities, resulting in poor correlation with the PRM data. 

As the main application of both methods is the quantitative comparison of the lysosomal proteome between different states, we performed a spike-in experiment to simulate constitutive upregulation of the whole lysosomal proteome and analyzed the sample by both PRM and DIA, applying 60 min gradients for both approaches. For this purpose, we combined LEF and LTL in a 1 to 5 ratio and compared the data to LTL samples without spike-in (Figure 1). In theory, as LEFs contain higher amounts of lysosomal proteins, this should result in a general increase of intensity for all lysosomal proteins present in the sample. 

For both approaches, the number of detected lysosomal proteins increased in comparison to LTL without spike-in, while the increase for PRM was 50% higher compared to DIA (223 to 243 for DIA and 278 to 308 for PRM, Figure 7b). Subsequently, we investigated the fold change ratios for proteins identified in all samples with both approaches (Figure 7c). We detected a median increase of intensity of 1.8 for PRM and 1.2 for DIA. When investigating values for individual proteins, we observed a discrepancy of ≥ 30% between fold change values acquired by DIA and PRM for 75% of proteins (average CV for DIA and PRM analysis of LTLs: 16% and 7%). While 142 proteins were detected with a higher value in PRM, only 35 were higher in the DIA data (Figure 7d). Classification of proteins based on their fold change values between the spike-in and the LTL sample further showed that DIA failed to detect any increase in signal intensity for 81 proteins upon spike-in, while this was only the case for 10 proteins in the PRM data (Figure 7e). Subsequently, we investigated if this effect was related to the abundance of individual proteins, as we observed markedly reduced correlation coefficients between DIA and PRM for lower abundant proteins in LTL samples (Figure 7a). Along this line, we grouped all proteins based on their abundance relative to the highest/lowest abundant protein in the respective dataset and plotted the observed fold change ratios for the individual groups (Figure 7f). While we observed highly similar fold change value distributions between LTL and spike-in samples for proteins across the whole range of abundance for the PRM data, a clear shift in the pattern of the DIA data was visible. Relative to the PRM data, DIA reported higher fold change ratios for low-abundant proteins while it resulted in lower values for high-abundant proteins. Taken together, these data indicate that PRM is better suited for the quantification of changes in the lysosomal proteome of LTL, which is mainly related to the better performance for the highest- and lowest-abundant lysosomal proteins in the dataset. 

## 4. Conclusions

In the present study, we analyzed the lysosomal proteome in samples of varying complexity by DIA and PRM. While both methods were well-suited for the analysis of lysosomal proteins in all samples, differences between the approaches became apparent that were mostly related to sample complexity. DIA identified more proteins in lower complexity samples and at longer gradients, since it was not limited by a predefined list of peptides, as was the case for the PRM analyses. Furthermore, no assay development was necessary for DIA analyses, thus greatly reducing the amount of time needed. For peptides covered by both approaches, DIA and PRM performed similarly for lower complexity LEFs, while PRM outperformed DIA in both MWCL and LTL. Especially for the quantification of protein level changes in LTL, PRM was able to identify significantly higher numbers of protein level alterations than DIA, which reported no change in abundance for a high number of proteins. Therefore, for the analysis of highly complex samples, such as whole tissue lysates, PRM presents the method of choice. Our developed PRM assay allows for the direct analysis of the lysosomal proteome from small amounts of whole tissue samples, without the need for lysosome enrichment, extending the toolbox for the investigation of the lysosomal proteome in complex samples.

## Data Availability

The mass spectrometry proteomics data have been deposited to the ProteomeXchange Consortium via the PRIDE [35] partner repository with the dataset identifier PXD023278.

## Supplementary Materials

The following are available online at https://www.mdpi.com/2227-7382/9/1/4/s1, Table S1: Reference list of high confidence lysosomal proteins and peptides covered by PRM assay. Supplementary Table S2: DIA Data, direct output from Spectronaut as well as processed data. Supplementary Table S3: PRM data, direct output from Skyline. Supplementary Table S4: PRM data filtered for dot p values > 0.7 and further analyses. Supplementary Figure S1: Dot product threshold determination for the acceptance of PRM data. Figure S2: Overlap of identified proteins from PRM and DIA runs.
