# Supplementary material for: Targeted Quantification of the Lysosomal Proteome in Complex Samples
Source: Proteomes. 2021 Jan 26;9(1):4. doi: 10.3390/proteomes9010004 (PMC7931001; doi:10.3390/proteomes9010004)
Supplement: Supplementary file 1 [file proteomes-09-00004-s001.zip › Supplementary_Figures.pdf]

Supplementary Material

Targeted Quantification of the Lysosomal Proteome in Complex Samples

Peter Mosen*, Anne Sanner*, Jasjot Singh, and Dominic Winter#

Institute for Biochemistry and Molecular Biology,
Medical Faculty, University of Bonn, 53115 Bonn, Germany

*these authors contributed equally

#Corresponding author
Dr. Dominic Winter
Institute for Biochemistry and Molecular Biology
Medical Faculty
Rheinische Friedrich-Wilhelms-University of Bonn
Nussallee 11, 53115 Bonn, Germany
Email: dominic.winter@uni-bonn.de
Tel: +49 228 73 7081

Supplementary Figure 1: Dot product threshold determination for the acceptance of PRM data.
Supplementary Figure 2: Overlap of identified proteins from PRM and DIA runs

Supplementary Reference

1. Hulsen, T., J. de Vlieg, and W. Alkema, BioVenn - a web application for the comparison and visualization of biological lists using area-proportional Venn diagrams. Bmc Genomics, 2008. 9(488).

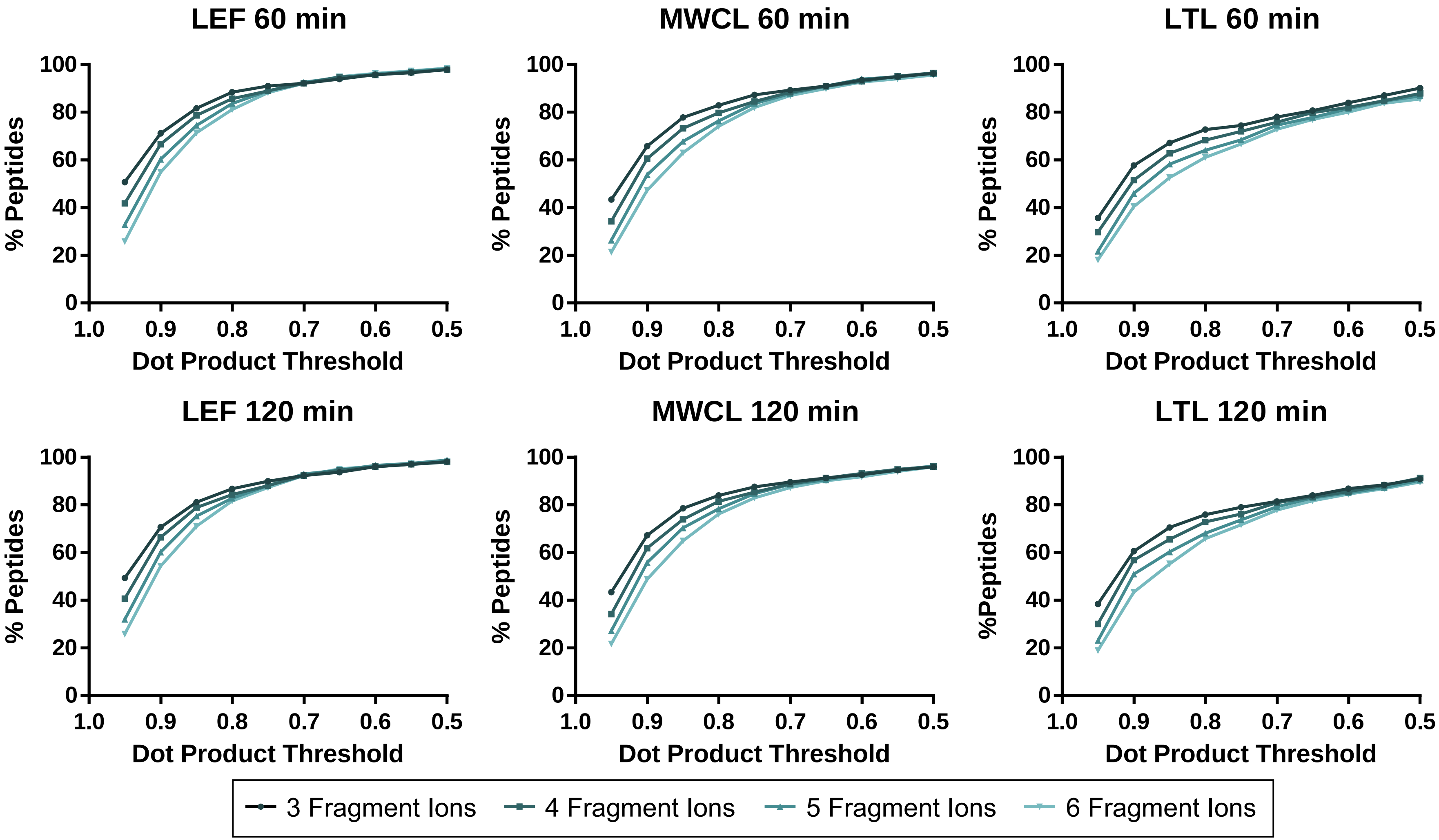

Supplementary Figure 1: Dot product threshold determination for the acceptance of PRM data. Shown are threshold determination graphs for 60 min and 120 min gradients for the lysosome-enriched fraction (LEF), mouse embryonic fibroblast whole cell lysate (MWCL), and liver tissue lysate (LTL). Data were analyzed with Skyline Daily using three, four, five, or six fragment ions. In each plot, the percentage of peptides passing the applied dot product threshold, which is indicated on the X axis, is displayed. Each line represents a different number of fragment ions utilized for peptide quantification.

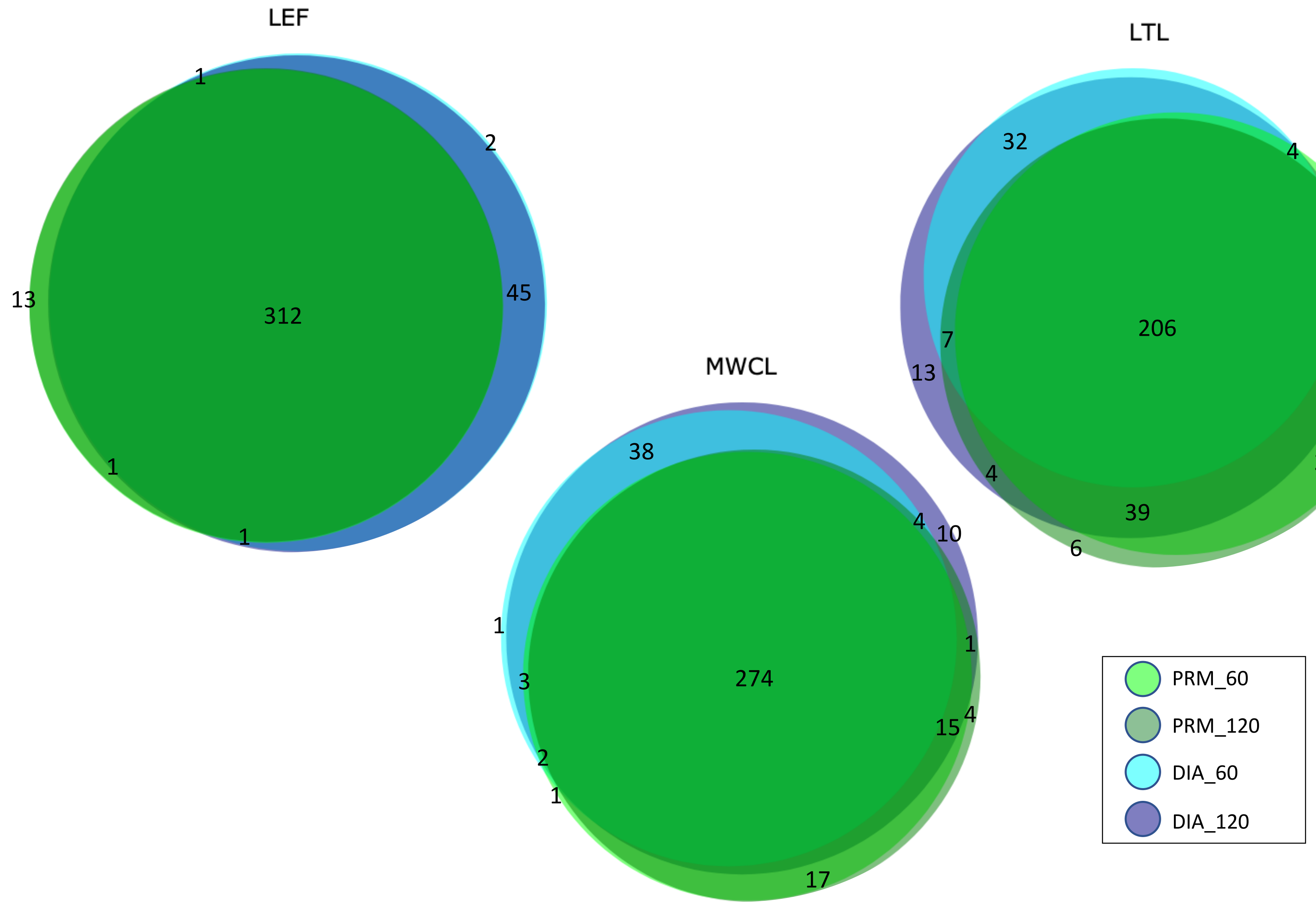

Supplementary Figure 2: Overlap of identified proteins from PRM and DIA runs. PRM data were analyzed using Skyline Daily, DIA data using Spectronaut. Proteins which were quantified in individual MS runs were extracted and compared. Each Venn diagram represents one sample type: lysosome-enriched fraction (LEF), mouse embryonic fibroblast whole cell lysate (MWCL), and liver tissue lysate (LTL). Compared are data from four different triplicate analyses conducted with PRM and DIA, each with 60 min and 120 min gradients. Venn diagrams were created using DeepVenn [1].
